# Fucoidan Isolated from *Sargassum confusum* Suppresses Inflammatory Responses and Oxidative Stress in TNF-α/IFN-γ- Stimulated HaCaT Keratinocytes by Activating Nrf2/HO-1 Signaling Pathway

**DOI:** 10.3390/md20020117

**Published:** 2022-02-01

**Authors:** Arachchige Maheshika Kumari Jayasinghe, Kirinde Gedara Isuru Sandanuwan Kirindage, Ilekuttige Priyan Shanura Fernando, Eui Jeong Han, Gun-Woo Oh, Won-Kyo Jung, Ginnae Ahn

**Affiliations:** 1 Department of Food Technology and Nutrition, Chonnam National University, Yeosu 59626, Korea; 218385@jnu.ac.kr (A.M.K.J.); 218388@jnu.ac.kr (K.G.I.S.K.); iosu5772@jnu.ac.kr (E.J.H.); 2 Department of Marine Bio-Food Sciences, Chonnam National University, Yeosu 59626, Korea; shanura@chonnam.ac.kr; 3 Research Center for Healthcare and Biomedical Engineering, Chonnam National University, Yeosu 59626, Korea; 4 Department of Biomedical Engineering and Center for Marine-Integrated Biomedical Technology (BK21 Plus), Pukyong National University, 45, Yongso-ro, Nam-gu, Busan 48513, Korea; oqwchobo@naver.com

**Keywords:** fucoidans, *Sargassum confusum*, inflammation, oxidative stress, HaCaT keratinocytes, (Nrf2)/(HO-1) signaling pathway

## Abstract

Recent studies have revealed that marine brown seaweeds contain numerous bioactive compounds which exhibit various bioactivities. The present study investigated the effect of low molecular weight fucoidan (SCF) isolated from *Sargassum confusum*, a brown alga, on inflammatory responses and oxidative stress in HaCaT keratinocytes stimulated by tumor necrosis factor (TNF)-α/interferon (IFN)-γ. SCF significantly increased the cell viability while decreasing the intracellular reactive oxygen species (ROS) production in TNF-α/IFN-γ-stimulated HaCaT keratinocytes. In addition, SCF effectively reduced inflammatory cytokines (interleukin (IL)-1β, IL-6, IL-8, IL-13, TNF-α, and IFN-γ) and chemokines (Eotaxin, macrophage-derived chemokine (MDC), regulated on activation, normal T cell expressed and secreted (RANTES), and thymus and activation-regulated chemokine (TARC)) expression, by down-regulating the expression of epithelial and epidermal innate cytokines (IL-25, IL-33, and thymic stromal lymphopoietin (TSLP)). Furthermore, SCF suppressed the activation of TNF-α/IFN-γ-stimulated mitogen-activated protein kinase (MAPK) and nuclear factor-κB (NF-κB) signaling pathways, while activating the nuclear factor erythroid 2-related factor 2 (Nrf2)/heme oxygenase-1 (HO-1) signaling pathway. The cytoprotective effect of SCF against TNF-α/IFN-γ stimulation was considerably reduced upon inhibition of HO-1 activity by ZnPP. Overall, these results suggest that SCF effectively suppressed inflammatory responses and oxidative stress in TNF-α/IFN-γ-stimulated HaCaT keratinocytes via activating the Nrf2/HO-1 signaling pathway.

## 1. Introduction

The skin, which is divided into two major compartments known as the dermis and the epidermis, is the primary interface between the internal body and the external environment, serving as the first line of defense against exogenous stimuli, such as microbial, chemical, and thermal agents [1,2]. HaCaT keratinocytes which are mainly present in the skin’s epidermis external layer are activated and produce inflammatory mediators that induce inflammation when exposed to inflammatory stimuli [3,4]. 

Inflammation is an important process for maintaining homeostasis in the body which is a part of the complex biological response of body tissues against harmful stimuli [5,6]. Nevertheless, inflammatory responses can be accompanied by oxidative stress, which may increase the risk of many diseases [7]. An increase in intracellular reactive oxygen species (ROS), which is thought to be the most potent inflammatory mediator, is defined as oxidative stress [8]. Uncontrolled inflammatory reactions take place to damage tissues and cause chronic inflammatory illnesses, such as atopic dermatitis, cardiovascular diseases, cancer, bronchitis, and asthma [5]. Tumor necrosis factor (TNF)-α and interferon (IFN)-γ are inflammatory mediators that promote oxidative stress and inflammatory reactions in HaCaT keratinocytes [9]. Exposure of the HaCaT keratinocytes to TNF-α and IFN-γ stimulates abnormal expression of cytokines, chemokines, and inflammatory mediated signaling pathways [10]. According to previous studies, TNF-α strongly induces regulated on activation, normal T cell expressed and secreted (RANTES) and interleukin (IL)-8 inflammatory mediators in addition to phosphorylation of mitogen-activated protein kinase (MAPK), while IFN-γ performs weak phosphorylation of MAP kinases and, with or without TNF-α, rapidly promotes the secretion of macrophage-derived chemokine (MDC) [11,12]. TNF-α or IFN-γ alone slightly induces thymus and activation-regulated chemokine (TARC) and MDC; nevertheless, when both were applied at the same time together, they were greatly stimulated [12,13,14]. The synergistic action of TNF-α and IFN-γ would be a strong driving factor of inflammation in a biological context. Therefore, those are widely used together to create disease models for assessing drug pharmacodynamic efficacy [1,5].

TNF-α/IFN-γ stimulation activates the nuclear transcription factor κB (NF-κB) pathway, which promotes phosphorylation of IκBα and NF-κB and allows the NF-κB p65 subunit to enter the nucleus from the cytosol, regulating the production of inflammatory mediators [9]. In addition, inflammatory responses are stimulated due to the activation of the MAPK pathway [5]. In HaCaT keratinocytes, TNF-α/IFN-γ-stimulated activation of NF-κB and MAPK signaling pathways induces the production of inflammatory cytokines (IL-1β, IL-6, IL-8, IL-13, TNF-α, and IFN-γ), and chemokines (Eotaxin, MDC, TARC, RANTES) in HaCaT keratinocytes [1,5,9,15]. The cytoprotective gene HO-1 is induced by activation of the nuclear factor erythroid 2-related factor 2 (Nrf2)/heme oxygenase-1 (HO-1) signaling pathway, which is mediated by Nrf2, and plays a crucial role in protecting cells against inflammatory reactions and oxidative damage [8,9]. Accordingly, inhibiting the expression of inflammatory mediators and oxidative stress may be important in the treatment of inflammatory skin conditions.

Natural resource-derived materials have recently piqued the interest of drug discovery researchers due to the availability of several bioactive compounds. Among them, marine-derived compounds are structurally diverse and have been reported to possess a wide range of biological actions [16,17]. Marine brown seaweeds contain a variety of bioactive compounds with varied bioactivities [5]. Fucoidan is a sulfated polysaccharide derived from marine brown algae that have antioxidant, anti-inflammatory, anti-obesity, anticancer, antitumor, antiviral, and antidiabetic properties [16,18,19,20]. The composition, source, molecular weight, purity level, and structure of fucoidan affect the features and efficacy of various biological activities, which can differ from species to species [21]. A previous study indicated that low MW fucoidan from *Sargassum coreanum* has great antioxidant capabilities that comprise its ultraviolet (UV)B-protective benefits [22]. Furthermore, substantial anti-photoaging and anti-melanogenesis properties of fucoidan from *Hizikia fusiforme* have been reported in an earlier study [23]. In addition, fucoidan is known for its anti-inflammatory and immunomodulatory effects [21]. Several prior studies have exhibited that fucoidan and fucoidan-containing extracts have anti-inflammatory activities in vitro and in vivo in several experimental models. The anti-inflammatory activity of *Sargassum horneri* fucoidan, as well as its protective benefits against fine dust-induced inflammation and deterioration of skin barrier functions in HaCaT keratinocytes, were examined in a prior study [24]. One of the previous in vitro investigations revealed that fucoidan isolated from *Padina commersonii* brown seaweed substantially reduced lipopolysaccharide (LPS)-induced inflammation in macrophages through downregulating the production of nitric oxide (NO), pro-inflammatory cytokines, and reducing activation of the NF-κB signaling pathway [25].

*Sargassum confusum* is one of the marine brown algae that is widely distributed along the coasts of China, Korea, and Japan which contains rich sulfated polysaccharides, such as fucoidans, amino acids, vitamins, dihomo-gammalinolenic acid, and trace elements, and possesses a wide range of biological activities [19,26]. In a recent study, the UVB protective effect of low molecular weight (MW) fucoidan isolated from *S. confusum* in HaCaT keratinocytes was investigated [26]. Nevertheless, there is no evidence of the anti-inflammatory and antioxidant effects of a fucoidan from *S. confusum* against TNF-α/IFN-γ stimulation. The current study was carried out by hypothesizing that low MW fucoidan of *S. confusum* (SCF) ameliorates inflammatory responses and oxidative stress in TNF-α/IFN-γ-stimulated HaCaT keratinocytes by suppressing the expression of inflammatory mediators and promoting the Nrf2/HO-1 signaling pathway.

## 2. Results

### 2.1. SCF Effectively Increases Cell Viability by Suppressing the Production of Intracellular ROS in TNF-α/IFN-γ-Stimulated HaCaT Keratinocytes

**Figure 1:**
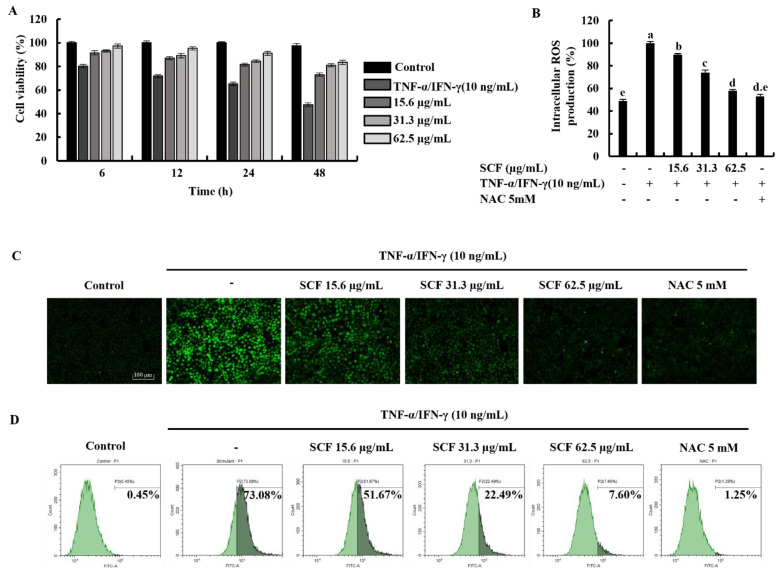
Protective effect of SCF in TNF-α/IFN-γ-stimulated HaCaT keratinocytes. (**A**) Time-dependent cell viability, (**B**) intracellular ROS generation, analysis of ROS level with and without TNF-α/IFN-γ stimulation by (**C**) fluorescence microscopy, and (**D**) flow cytometer. For ROS assays, cells were treated with DCF-DA. The results represent data from three independent experiments (*n* = 3), and values are indicated as the means ± SE. Error bars with different letters are significantly different (*p* ˂ 0.05).

Time-dependent cell viability for 15.6, 31.3, and 62.5 µg/mL SCF concentrations with TNF-α/IFN-γ-stimulation was measured using MTT assay. As shown in Figure 1A, TNF-α/IFN-γ stimulation decreased cell viability in HaCaT keratinocytes, whereas SCF treatment resulted in a dose-dependent increase in cell viability. At a given concentration, it was observed that there was a gradual decrease in the value of the cell viability over time. All SCF concentrations showed a uniform pattern of change in cell viability over time. The results of the DCF-DA assay using a microplate reader indicated an increment in intracellular ROS levels in TNF-α/IFN-γ-stimulated HaCaT keratinocytes (Figure 1B), while significantly decreasing with SCF treatment dose-dependently. As depicted in Figure 1C, TNF-α/IFN-γ-stimulated HaCaT keratinocytes produced high fluorescence, indicating an increase in intracellular ROS levels, whereas SCF treatment resulted in a dose-dependent decrease in fluorescence intensity. Furthermore, flow cytometric analysis of DCF-DA stained cells revealed an increase in intracellular ROS levels in TNF-α/IFN-γ-stimulated HaCaT keratinocytes (Figure 1D), which was diminished in a dose-dependent manner with SCF treatment. N-acetylcysteine (NAC) was used as a positive control for ROS scavenging. 

### 2.2. SCF Downregulates the Expression of Inflammatory Cytokines and Chemokines in TNF-α/IFN-γ-Stimulated HaCaT Keratinocytes

**Figure 2:**
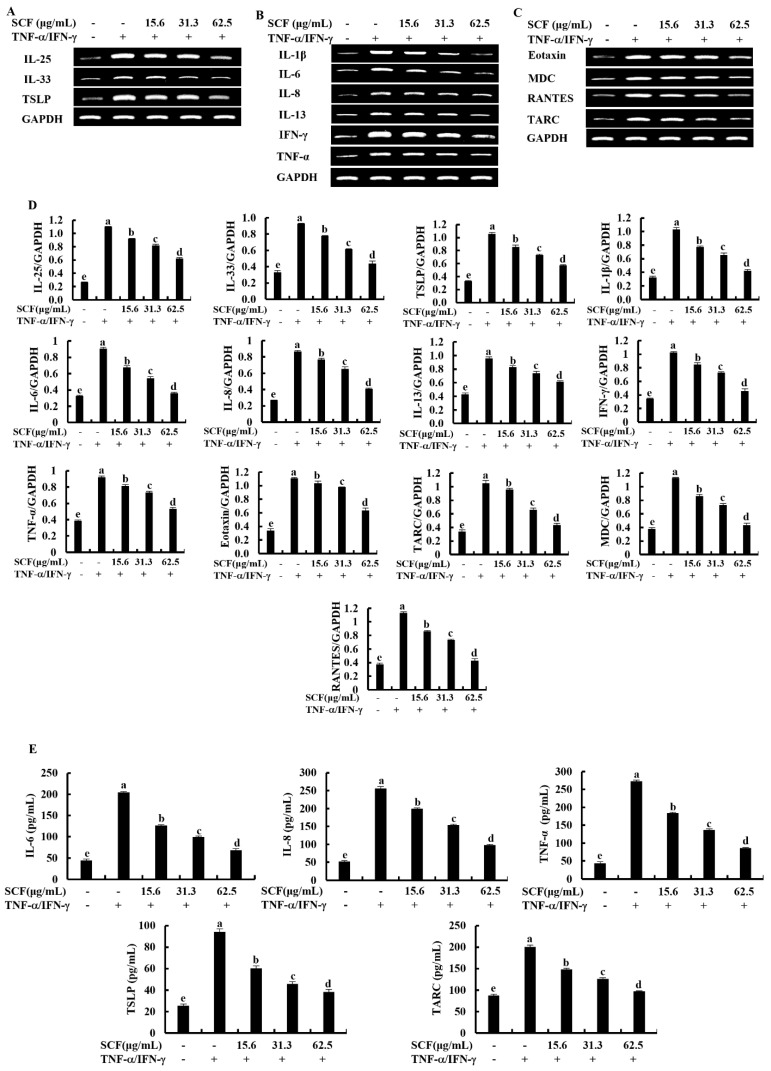
Inhibitory effect of SCF on mRNA expression of inflammatory cytokines and chemokines. Effect of SCF against the mRNA expression of (**A**) epithelial and epidermal innate cytokines, (**B**) inflammatory cytokines, (**C**) chemokines, (**D**) densitometry analysis, and (**E**) ELISA analysis of cytokines and chemokines production in TNF-α/IFN-γ-stimulated HaCaT keratinocytes. The reproducibility of the results was confirmed by three independent determinant determinations (*n* = 3, mean ± SE). Error bars with different letters show a significant difference (*p* ˂ 0.05).

The expression levels of key inflammatory cytokines and chemokines were evaluated by Reverse Transcription Polymerase Chain Reaction (RT-PCR) and Enzyme-linked immunosorbent assay (ELISA) analysis. According to Figure 2A, TNF-α/IFN-γ stimulation upregulated mRNA expression levels of epithelial and epidermal innate cytokines, including IL-25, IL-33, and TSLP, compared to the control group while downregulated by SCF treatment markedly. Stimulation of TNF-α/IFN-γ upregulated the mRNA expression levels of IL-1β, IL-6, IL-8, IL-13, TNF-α, and IFN-γ inflammatory cytokines (Figure 2B), and Eotaxin, MDC, RANTES, and TARC chemokines (Figure 2C) in HaCaT keratinocytes, whereas SCF treatment markedly suppressed the mRNA expression levels of inflammatory cytokines and chemokines in TNF-α/IFN-γ-stimulated HaCaT keratinocytes. Densitometry analysis of inflammatory cytokines and chemokines expression was indicated by Figure 2D. According to the ELISA analysis, SCF dose-dependently reduced IL-6, IL-8, TNF-α, TSLP, and TARC production in TNF-α/IFN-γ-stimulated HaCaT keratinocytes (Figure 2E).

### 2.3. SCF Suppresses the Activation of MAPK and NF-κB Signaling Pathways in TNF-α/IFN-γ-Stimulated HaCaT Keratinocytes

**Figure 3:**
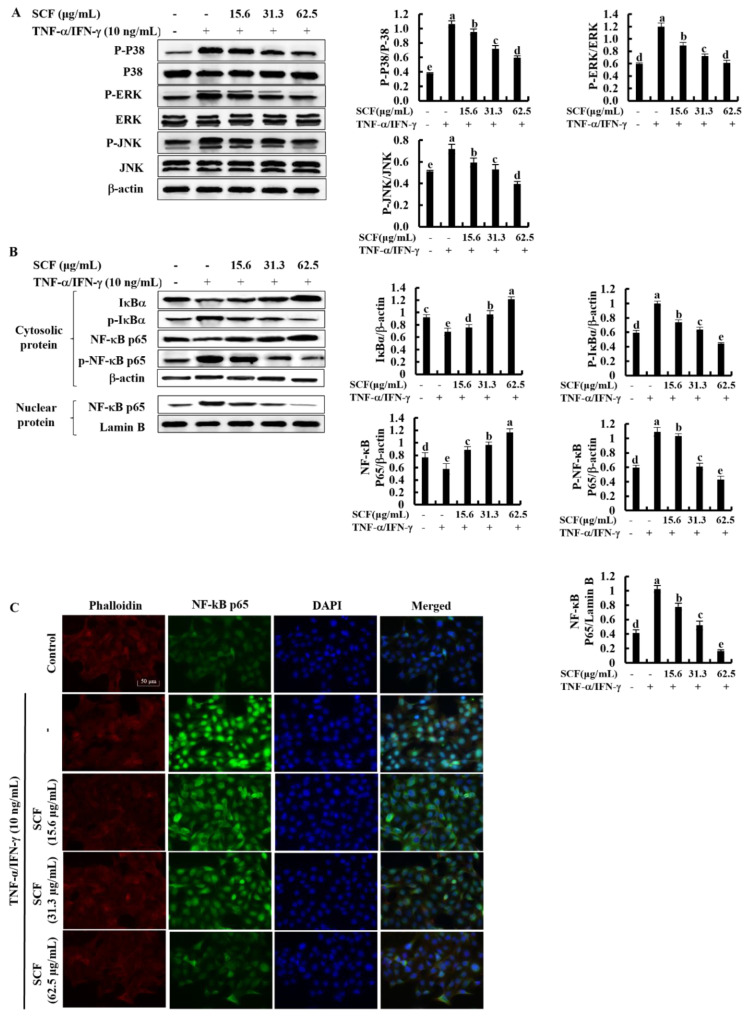
Inhibitory effect of SCF on MAPK and NF-κB molecular mediators in TNF-α/IFN-γ-stimulated HaCaT keratinocytes. (**A**) Western blot analysis of key MAPK mediators, (**B**) phosphorylation and nuclear translocation of NF-κB molecular mediators. (**C**) Immunofluorescence analysis of NF-κB p65 nuclear translocation. All experiments were performed in triplicate (*n* = 3), and values are indicated as the means ± SE. Error bars with different letters show a significant difference (*p* ˂ 0.05).

The NF-κB and MAPK signaling pathways are critical in upstream interconnected signaling pathways that are responsible for inflammatory responses [27]. Based on Western blot analysis, TNF-α/IFN-γ stimulation significantly increased the phosphorylation of p38, ERK, and JNK MAPK mediators compared to the control while decreasing with SCF treatment dose-dependently (Figure 3A). Besides, phosphorylation of NF-κB mediators, including cytosolic IκBα and NF-κB p65, increased by TNF-α/IFN-γ stimulation while increasing nuclear translocation of NF-κB p65 (Figure 3B). Nevertheless, SCF treatment considerably reduced phosphorylation of NF-κB mediators as well as nuclear translocation of the NF-κB p65 dose-dependent manner. Similarly, nuclear translocation of NF-κB p65 was evaluated by immunofluorescence analysis (Figure 3C). The presence of green fluorescence in the nucleus can be used to identify NF-κB p65 nuclear translocation. The increased nuclear translocation of NF-κB p65 is indicated by the bright green fluorescent signals. According to the immunofluorescence analysis, TNF-α/IFN-γ-stimulation increased nuclear translocation of NF-κB p65 compared to the control, while treatment of SCF attenuated it dose-dependently.

### 2.4. SCF Activates the Nrf2/HO-1 Signaling Pathway in TNF-α/IFN-γ-Stimulated HaCaT Keratinocytes

**Figure 4:**
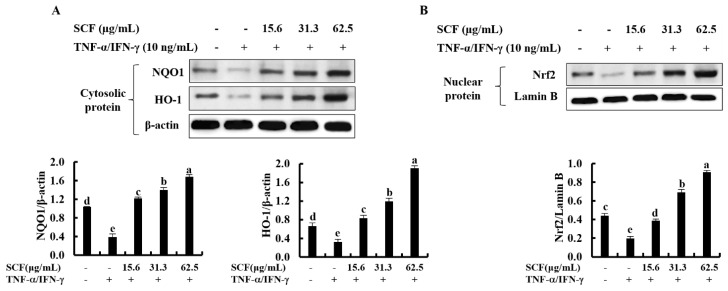
Cytoprotective effect of SCF on the activation of Nrf2/HO-1 signaling pathway. (**A**) Dose-dependent response of the SCF on activation of HO-1 and NQO1, and (**B**) nuclear-translocated Nrf2 in TNF-α/IFN-γ-stimulated HaCaT keratinocytes by Western blot analysis. Data are represented as mean ± SE of three independent determinations (*n* = 3). Mean values with different letters are significantly different (*p* ˂ 0.05).

The Nrf2/HO-1 signaling pathway is a key mediator in protecting cells from oxidative damage [28]. To assess whether SCF affects the Nrf2/HO-1 signaling pathway, levels of cytosolic HO-1, NQO1, and nuclear-translocated Nrf2 were investigated using Western blot analysis. As shown in Figure 4A, cytosolic HO-1 and NQO1 were down-regulated following cellular stimulation with TNF-α/IFN-γ, while significantly increased by the SCF treatment dose-dependently. Moreover, TNF-α/IFN-γ stimulation decreased nuclear translocation of Nrf2, while SCF treatment dose-dependently increased nuclear Nrf2 expression compared to TNF-α/IFN-γ-stimulated HaCaT keratinocytes, revealing its cytoprotective effect (Figure 4B).

### 2.5. Effect of the ZnPP in TNF-α/IFN-γ-Stimulated HaCaT Keratinocytes with the Treatment of SCF

**Figure 5:**
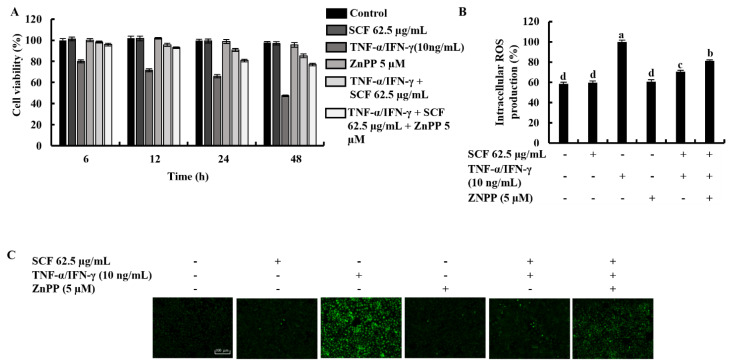
Effect of HO-1 inhibition by ZnPP on SCF treated (**A**) time-dependent cell viability, (**B**) intracellular ROS production, and (**C**) analysis of ROS levels by fluorescence microscopy in TNF-α/IFN-γ-stimulated HaCaT keratinocytes. The results represent data from three independent experiments (*n* = 3), and values are indicated as the means ± SE. Error bars with different letters show a significant difference (*p* ˂ 0.05).

The cytoprotective effect of SCF on TNF-α/IFN-γ-stimulated HaCaT keratinocytes was investigated concerning variations in time-dependent cell viability and ROS production in the presence of ZnPP by inhibiting HO-1 activity. As shown in Figure 5A,B, TNF-α/IFN-γ stimulation significantly decreased cell viability and increased intracellular ROS production, while SCF treatment effectively increased cell viability by reducing the production of ROS dose-dependent manner. Nevertheless, the HO-1 inhibitor ZnPP significantly abolished the increment of cell viability and caused a reduction of intracellular ROS production. Similarly, the effect of the ZnPP on intracellular ROS production in SCF treated TNF-α/IFN-γ-stimulated HaCaT keratinocytes was investigated by fluorescence microscopy DCF-DA assay (Figure 5C). Besides, the effect of SCF on cell viability in TNF-α/IFN-γ-stimulated HaCaT keratinocytes gradually decreased with time. As expected, ZnPP reduced the cell viability which had been increased by SCF over time.

## 3. Discussion

Various kinds of seaweeds have been used in traditional medicine in the Asian region due to their excellent characteristics for centuries and have recently gained significant interest as a supplement in the functional food industry due to their availability and functional properties [25]. Brown algae are rich in bioactive compounds which produce numerous secondary metabolites with interesting biological activities, such as polysaccharides, phlorotannin, and diterpene [29]. Fucoidan is a sulfated polysaccharide found in the cell walls of several types of brown algae [30]. Numerous protective effects of fucoidan isolated from various brown algae have been reported in previous studies [16,20,25]. One of the prior investigations examined the effects of fucoidan on reducing apoptosis-related processes comprising apoptotic body formation and DNA damage, demonstrating its UVB protective ability. Fucoidan treatment suppressed the changes in important skin barrier proteins and molecular mediators that preserve integrity and moisturization in UVB-stimulated cells [26]. Increased production of NO, inducible forms of nitric oxide synthase (iNOS), cyclooxygenase-2 (COX-2), prostaglandin E2 (PGE_2_), and inflammatory cytokines, such as TNF-α, IL-1β, and IL-6, cause inflammation in macrophages as a result of LPS stimulation. In a dose-dependent way, treatment of fucoidan substantially inhibited NO production and expression of iNOS, COX-2, PGE_2_, and proinflammatory cytokines [25]. Numerous studies have shown that low MW fucoidans with a higher degree of sulfation reduce intracellular ROS levels and inhibit NF-kB and MAPK inflammatory mediated pathways, which regulate gene expression of inflammatory cytokines and chemokines that cause inflammation [18,24,26]. In addition, the protective effects of low MW fucoidan isolated from *S. confusum* (SCF) against inflammatory responses and oxidative stress in TNF-α/IFN-γ-stimulated HaCaT keratinocytes were investigated in this study. 

HaCaT keratinocytes are capable of producing cytokines and chemokines which contribute to initiating and regulating cutaneous inflammatory reactions [31]. A range of concentrations (15.6, 31.3, and 62.5 µg/mL) of SCF were used for further studies. One of the key factors that contribute to inflammatory disorders is oxidative stress, which is caused by the overproduction and accumulation of ROS in live cells. Generation of ROS reduces with fucoidan due to the immunomodulatory effects of sulfated polysaccharides [26]. According to the findings, SCF dose-dependently increased the cell viability, whereas a decrease in intracellular ROS production in TNF-α/IFN-γ-stimulated HaCaT keratinocytes. The cell viability of all SCF concentrations changed in a consistent pattern throughout time. The protective effect of SCF was further confirmed by fluorescence microscopy and flow cytometry analysis of DCF-DA stained cells. In a nutshell, the above results indicated that SCF possesses a considerable protective effect against oxidative stress by decreasing the production of ROS. 

TNF-α/IFN-γ-stimulated HaCaT keratinocytes can cause the expression of inflammatory mediators such as cytokines, chemokines, and adhesion molecules, leading to skin inflammation [1,9]. In the current study, RT-PCR and ELISA analysis confirmed the effect of SCF on the mRNA expression levels of inflammatory mediators. The mRNA expression levels of IL-25, IL-33, and TSLP significantly increased in TNF-α/IFN-γ-stimulated HaCaT keratinocytes compared to the control group, same as reported in previous studies [5,17]. IL-25, IL-33, and TSLP are epithelial and epidermal innate cytokines that play an important role in the initiation of inflammatory disorders by regulating the production of cytokines [32]. Inflammatory cytokines and chemokines are produced predominantly by activated HaCaT keratinocytes and are conducive to mediating inflammatory responses [33]. TNF-α/IFN-γ-stimulated HaCaT keratinocytes induce the expression of inflammatory cytokines IL-1β, IL-6, IL-8, IL-13, TNF-α, IFN-γ, and chemokines Eotaxin, MDC, RANTES, and TARC [1,9]. The most well-known mediators in inflammatory cells migration, keratinocyte proliferation, and the production of additional cytokines by keratinocytes are IL-1β and IL-6 [34]. Chemokines are small proteins that are secreted by a variety of cell types and influence immune cell movement to the site of inflammation or infection [35]. TARC, MDC, and RANTES are typical inflammatory chemokines that bind to CC chemokine receptor 4 (CCR4), which is found mainly on keratinocytes [35]. Another inflammatory mediator involved in allergic responses and linked to the severity of chronic inflammation is IL-8 [36]. In the current study, expression of inflammatory cytokines IL-1β, IL-6, IL-8, IL-13, TNF-α, IFN-γ, and chemokines Eotaxin, MDC, RANTES, TARC were increased in TNF-α/IFN-γ-stimulated HaCaT keratinocytes compared to the control and markedly suppressed by SCF treatment. The cumulative expression of the above results confirmed the effect of SCF on TNF-α/IFN-γ-stimulated inflammation in HaCaT keratinocytes. The protective effect of the SCF against the production of inflammatory mediators was further confirmed by the results of the ELISA analysis.

Multiple signaling pathways comprising NF-κB and MAPK interact with the inflammatory responses in HaCaT keratinocytes via activating and producing inflammatory mediators [24]. The NF-κB pathway is interconnected with the MAPK signaling pathway, which mediates phosphorylation processes that lead to the activation of several transcription factors involved in inflammation [9]. These factors, together with NF-kB, activate the transcription of different inflammatory genes, including those encoding for the inflammatory mediators [6]. In the present study, the expression of phosphorylated p38, ERK, and JNK MAPK mediators was significantly increased in TNF-α/IFN-γ-stimulated HaCaT keratinocytes. However, reduction of inflammatory responses through decreasing phosphorylation of MAPK mediators has been reported in previous studies [15]. Similarly, the treatment of SCF significantly reduced the activation of the MAPK pathway stimulated by TNF-α/IFN-γ dose-dependently. In the cytosol, the most prominent NF-κB dimer is found in an inactive state bound to IκBα, a natural NF-κB inhibitor. Activation of NF-κB, involving phosphorylation and release of IκBα followed by NF-κB p65 nuclear translocation, is induced by harmful inflammatory stimuli [37]. The expression of different inflammatory mediated genes was increased followed by translocation of NF-κB p65 into the nucleus [38]. As results obtained in the present study show, phosphorylation of cytosolic IκBα, NF-κB p65 and nuclear translocation of NF-κB p65 in TNF-α/IFN-γ-stimulated HaCaT keratinocytes were increased while effectively suppressed by SCF treatment, dose-dependently evidenced by both Western blot and immunofluorescence analysis.

The activation of the Nrf2/HO-1 signaling pathway in HaCaT keratinocytes is a well-known cytoprotective cellular mechanism that involves curing various diseases related to oxidative stress [8,39]. Nrf2 typically interacts with a cytosolic actin-binding protein called Keap1(Kelch-like ECH-associated protein 1), an inhibitor of Nrf2 [40]. Nrf2 dissociates from Keap1, becomes stabilized, and translocates into the nucleus under the conditions of oxidative stress. Following that, it activates the transcription of numerous antioxidant genes, including NQO1 and HO-1 [26]. HO-1 is an inducible cytoprotective enzyme that effectively downregulates the expression of molecules related to the development of oxidative stress and inflammatory responses in the range of cell lines [8,26]. The current study investigated the capability of SCF to stimulate the activation and nuclear translocation of Nrf2 as well as the activation of NQO1 and HO-1 antioxidant gene transcription. The results of this study indicated that expression of HO-1 and NQO1 found in the cytosol and nuclear-translocated Nrf2 was decreased in TNF-α/IFN-γ-stimulated HaCaT keratinocytes. Conversely, the antioxidant potential of SCF dose-dependently increased the activation of the Nrf2/HO-1 signaling pathway. Furthermore, we investigated the effect of ZnPP (HO-1 inhibitor) on the inhibitory effect of ROS production by SCF in TNF-α/IFN-γ-stimulated HaCaT keratinocytes and examined that ZnPP weakened the ROS scavenging activity of SCF [41]. These findings indicated that the protective effect of SCF on TNF-α/IFN-γ-stimulated oxidative stress and inflammatory responses in HaCaT keratinocytes is mediated by activation of the Nrf2/HO-1 signaling pathway and that the protective role of SCF on TNF-α/IFN-γ-stimulated HaCaT keratinocytes was dependent on HO-1.

## 4. Materials and Methods

### 4.1. Materials

The low MW fucoidan derived from *S. confusum* (SCF) was obtained during the previous study [26]. Dulbecco’s Modified Eagle Medium (DMEM), and penicillin/streptomycin mixture were purchased from GibcoBRL (Grand Island, NY, USA). Fetal bovine serum (FBS) was obtained from Welgene (Gyeongsangbuk-do, South Korea). Recombinant TNF-α and IFN-γ were purchased from R&D Systems (Minneapolis, MN, USA). The 3-(4,5-dimethylthiazol-2-yl)-2,5-diphenyltetrazolium bromide (MTT), 2´,7´-Dichlorofluorescin diacetate (DCF-DA), dimethyl sulfoxide (DMSO), bovine serum albumin (BSA), ethidium bromide, agarose, Triton^TM^ X-100, paraformaldehyde, TRIzol, chloroform, isopropanol, and zinc protoporphyrin IX (ZnPP) were purchased from Sigma-Aldrich (St. Louis, MO, USA). BCA protein assay kit, NE-PER^®^ nuclear and cytoplasmic extraction kit, 1-Step transfer buffer, Pierce™ RIPA buffer, protein ladder, and diethyl pyrocarbonate (DEPC) water were obtained from Thermo Fisher Scientific (Rockford, IL, USA). Ace-α-® cDNA synthesis kit was purchased from ReverTra (Toyobo, Osaka, Japan). Skim milk powder was obtained from BD Difco™ (Sparks, MD, USA). Primary and secondary antibodies, Prolong^®^ Gold antifade reagent with DAPI, normal goat serum, and DyLightTM 554 Phalloidin were obtained from Cell Signaling Technology (Beverly, MA, USA). The primers for reverse transcription-polymerase chain reaction (RT-PCR) were purchased from Bioneer Co. (Deadeock-gu, Daejeon, Korea). ELISA kits for human IL-6, IL-8, TNF-α, TSLP, and TARC were obtained from BioLegend (San Diego, CA, USA).

### 4.2. Cell Culture and SCF Treatment

HaCaT keratinocytes purchased from the Korean Cell Line Bank (Seoul, Korea) were cultured at 37 °C in an incubator with a humidified environment of 5% CO_2_ in DMEM medium supplemented with 10% FBS and 1% penicillin/streptomycin mixture. Once every two days, cells were sub-cultured until an exponential growth was observed that was suitable for seeding. SCF was diluted with PBS before use in the experiments to adjust the final treatment concentrations.

### 4.3. Cell Viability Assay

To evaluate cell viability, the MTT assay was used [42]. In brief, cells were seeded in 96-well plates at a density of 1 × 10^4^ cells per well and incubated for 24 h. Cells were treated for 1 h with different concentrations of SCF ranging from 15.6–62.5 µg/mL before being stimulated with TNF-α and IFN-γ mixture ratio 1:1. After 24 h of TNF-α/IFN-γ stimulation, 15 µL of MTT reagent (5 mg/mL) was added to cells and incubated for 4 h at 37 °C. After removing the medium, the formed formazan crystals were dissolved in DMSO for 30 min. Absorbance was measured at 570 nm using a SpectraMax M2 microplate reader (Molecular Devices, Silicon Valley, CA, USA).

### 4.4. Analysis of Intracellular ROS Production

The effect of SCF on intracellular ROS production in TNF-α/IFN-γ-stimulated HaCaT keratinocytes was investigated using the DCF-DA assay. After 24 h of 1 × 10^5^ cells/well were seeded in 24 well plates, treated with SCF sample concentrations of 15.6, 31.3, and 62.5 μg/mL, and incubated for 1 h before stimulation with TNF-α/IFN-γ. After 1 h incubation, DCF-DA was added to cells and intracellular ROS production was measured. For fluorometric analysis, a SpectraMax M2 microplate reader was used with excitation and emission wavelengths of 485 and 528 nm, respectively. Aside from fluorometry, the DCF DA-treated cells were examined using a Thermo Fisher Scientific EVOS M5000 Imaging fluorescent microscope (Rockford, IL, USA) and a CytoFLEX flow cytometer (Beckman Coulter, Brea, CA, USA).

### 4.5. Western Blot Analysis

A total of 1 × 10^6^ cells/dish were seeded in 6 cm culture dishes for 24 h and treated with 15.6, 31.3, and 62.5 μg/mL of SCF treatment before TNF-α/IFN-γ stimulation. After that, cells were harvested and lysed using the NE-PER^®^ nuclear and cytoplasmic extraction kit. Totals of 30 µg of protein of each lysate were electrophoresed on 10% polyacrylamide gels after protein concentrations in cell lysate were estimated using a BCA protein assay kit. Resolved protein bands were transferred onto nitrocellulose membranes (Merck Millipore, Ireland), and blocked for 2 h with 5% skim milk in TBST. Then, sequentially incubated with monoclonal primary antibodies (1:1000) and HRP-conjugated secondary antibodies (1:3000). Identified protein bands were visualized by adding enhanced chemiluminescence (ECL) reagent on a Core Bio Davinch-ChemiTM imaging system (Seoul, Korea).

### 4.6. RNA Extraction and RT-PCR Analysis

The cells were seeded in 6 cm culture dishes for 24 h and treated with different doses of SCF before TNF-α/IFN-γ stimulation. This was followed by cell collection and homogenization with TRIzol reagent. The homogenized cells were treated with chloroform to separate the phases and centrifuged at 12,000 rpm for 15 min at 4 °C. The upper layer of supernatant was collected and mixed with isopropanol, then centrifuged for 15 min at 15,000× *g* at 4 °C. To obtain total RNA, the supernatant was removed, and the pellet was dissolved in DEPC water. The ReverTra Ace-α-^®^ cDNA synthesis kit was used to synthesize cDNA from total RNA according to the manufacturer’s instructions. RT-PCR was performed on the prepared cDNA using the relevant primers. In a TaKaRa PCR Thermal Cycler (TaKaRa Bio Inc., Otsu, Japan), 30 PCR cycles of denaturation at 94 °C for 1 min, annealing at 55–60 °C for 1 min, and extension at 72 °C for 20 min, were performed. After 0.5 µg/mL ethidium–bromide treatment, the amplified PCR products were electrophoresed for 30 min at 100 V on 1.5% agarose gel and visualized under UV illumination. ImageJ software (Version 1.52a, US National Institutes of Health, Bethesda, MD, USA) was used to quantify the relative intensities of expression levels, which were normalized with GAPDH.

### 4.7. ELISA Analysis

After 24 h of cell seeding, cells were treated for 1 h with different SCF concentrations before being stimulated with TNF-α/IFN-γ for 24 h. The supernatants were collected in the multi-well plates using the interchangeable centrifuge rotor (400× *g*, Hanil Science Industrial, Incheon, Korea). The production of IL-6, IL-8, TNF-α, TSLP, and TARC were then evaluated using the ELISA assay kits according to the manufacturer’s instructions. In brief, the sample was added to the coated plate and incubated for 2 h before being washed 5 times with 1× washing buffer. It was immediately treated with the detection antibody, and after 1 h, Avidin-HRP buffer was added and incubated for 30 min. The stop solution was added to the plates and measured at 450 nm with SpectraMax M2 microplate reader [5].

### 4.8. Immunofluorescence Analysis

The analysis was conducted following the previous study [26]. In brief, 1 × 10^4^ cells/well were seeded in eight well chamber slides and incubated for 24 h in a humidified environment before SCF sample treatment. Wells were rinsed with PBS and fixed with 4% formaldehyde after 30 min of TNF-α/IFN-γ stimulation. After PBS washing, cells were incubated for 1 h in blocking buffer (PBS containing 5% normal goat serum and 0.3% Triton^TM^ X-100). The wells were treated for 2 h with Alexa Fluor® 488 conjugated Anti-Mouse IgG after being incubated overnight with a primary antibody (anti-NF-kB p65). Followed by PBS washing, slides were covered with coverslips using Prolong^®^ Gold antifade reagent with DAPI, and cells were visualized using a Thermo Fisher Scientific EVOS M5000 Imaging fluorescence microscope.

### 4.9. Statistical Analysis

All data were expressed as the mean ± standard error (SE) of at least three independent determinants. Statistical comparisons were carried out by one-way analysis of variance (ANOVA) followed by Duncan’s multiple range test using IBM SPSS software (Version 24.0, Chicago, IL, USA), and *p* ˂ 0.05 was considered to indicate statistical significance.

## 5. Conclusions

In summary, the present study revealed that SCF effectively suppressed the oxidative stress and the inflammatory responses in TNF-α/IFN-γ-stimulated HaCaT keratinocytes by reducing the expression of inflammatory mediators through regulation of NF-κB and MAPKs signaling pathways via activating Nrf2/HO-1 signaling.

## Data Availability

The data presented in this study are available on request from the corresponding author.
